# Comparing the reliability of muscle oxygen saturation with common performance and physiological markers across cycling exercise intensity

**DOI:** 10.3389/fspor.2023.1143393

**Published:** 2023-08-04

**Authors:** Assaf Yogev, Jem Arnold, Hannah Nelson, David C. Clarke, Jordan A. Guenette, Ben C. Sporer, Michael S. Koehle

**Affiliations:** ^1^ Environmental Physiology Laboratory, The University of British Columbia, School of Kinesiology, Vancouver, BC, Canada; ^2^ Department of Biomedical Physiology and Kinesiology and Sports Analytics Group, Simon Fraser University, Burnaby, BC, Canada; ^3^ Deptartment of Physical Therapy, The University of British Columbia, Vancouver, BC, Canada; ^4^ Centre for Heart Lung Innovation, Providence Research, The University of British Columbia and St. Paul’s Hospital, Vancouver, BC, Canada; ^5^ Department of Family Practice, Vancouver Whitecaps FC, Vancouver, BC, Canada; ^6^ Division of Sport & Exercise Medicine, The University of British Columbia, Vancouver, BC, Canada

**Keywords:** muscle oxygen saturation, near-infrared spectroscopy, repeatability, metabolic analysis, incremental exercise test, wearable, exercise testing, training zones

## Abstract

**Introduction:**

Wearable near-infrared spectroscopy (NIRS) measurements of muscle oxygen saturation (SmO_2_) demonstrated good test–retest reliability at rest. We hypothesized SmO_2_ measured with the Moxy monitor at the vastus lateralis (VL) would demonstrate good reliability across intensities. For relative reliability, SmO_2_ will be lower than volume of oxygen consumption (V̇O_2_) and heart rate (HR), higher than concentration of blood lactate accumulation ([BLa]) and rating of perceived exertion (RPE). We aimed to estimate the reliability of SmO_2_ and common physiological measures across exercise intensities, as well as to quantify within-participant agreement between sessions.

**Methods:**

Twenty-one trained cyclists completed two trials of an incremental multi-stage cycling test with 5 min constant workload steps starting at 1.0 watt per kg bodyweight (W·kg^−1^) and increasing by 0.5 W kg^−1^ per step, separated by 1 min passive recovery intervals until maximal task tolerance. SmO_2_, HR, V̇O_2_, [BLa], and RPE were recorded for each stage. Continuous measures were averaged over the final 60 s of each stage. Relative reliability at the lowest, median, and highest work stages was quantified as intraclass correlation coefficient (ICC). Absolute reliability and within-subject agreement were quantified as standard error of the measurement (SEM) and minimum detectable change (MDC).

**Results:**

Comparisons between trials showed no significant differences within each exercise intensity for all outcome variables. ICC for SmO_2_ was 0.81–0.90 across exercise intensity. ICC for HR, V̇O_2_, [BLa], and RPE were 0.87–0.92, 0.73–0.97, 0.44–0.74, 0.29–0.70, respectively. SEM (95% CI) for SmO_2_ was 5 (3–7), 6 (4–9), and 7 (5–10)%, and MDC was 12%, 16%, and 18%.

**Discussion:**

Our results demonstrate good-to-excellent test-retest reliability for SmO_2_ across intensity during an incremental multi-stage cycling test. V̇O_2_ and HR had excellent reliability, higher than SmO_2_. [BLa] and RPE had lower reliability than SmO_2_. Muscle oxygen saturation measured by wearable NIRS was found to have similar reliability to V̇O_2_ and HR, and higher than [BLa] and RPE across exercise intensity, suggesting that it is appropriate for everyday use as a non-invasive method of monitoring internal load alongside other metrics.

## 1. Background

Muscle oxygenation is becoming an increasingly popular physiological marker of the internal load to exercise for athletes, practitioners, and sport scientists (1). With the introduction of wearable near-infrared spectroscopy (NIRS), the potential to monitor muscle oxygen saturation (SmO_2_) in real-time at the location where the activity occurs is becoming increasingly viable. For measurements intended to be used in everyday training environments, the reliability between sessions, i.e., test–retest reliability is a necessary step before use. Despite its importance, deciphering what change over time is expected in common physiological markers remains challenging. Part of this challenge is due to the variety of ways reliability can be assessed using the same measure for different sporting applications (2).

Both relative and absolute test–retest reliability of muscle oxygenation signals measured by the Moxy muscle oxygen monitor (Fortiori Design LLC., Hutchinson, MN, USA), in particular muscle oxygen saturation, has been investigated in the conditions of rest, vascular occlusion, and exercise (3–5). Relative reliability evaluates the observed measurement error relative to observed differences between participants and relates to the ability to consistently differentiate participants. Absolute reliability evaluates the consistency of repeated measurements (repeatability) within a single individual (2, 6). Agreement evaluates how close any two measurements are, in the original units of the parameter being evaluated (2, 6). For this sensor, comparing the reliability of SmO_2_ with other common physiological measures such as heart rate (HR), rate of systemic oxygen uptake (V̇O_2_), concentration of blood lactate ([BLa]), and rating of perceived exertion (RPE), will provide a useful contrast for each of these common markers during the same exercise protocol.

The behaviour of SmO_2_ has been studied in both incremental ramp exercise and constant workloads (CW) protocols at various intensities (7–14). SmO_2_ is a measure of the relative concentration of oxygenated and deoxygenated hemoglobin and myoglobin in the tissue under the sensor, and reflects the balance of local O_2_ delivery and O_2_ extraction (i.e., O_2_ supply to match energetic demand) (3, 4, 15). A higher rate of O_2_ delivery than extraction, all else equal, will tend to produce rising SmO_2_ (excess supply). A higher rate of O_2_ extraction over delivery will produce falling SmO_2_ (excess demand). At higher exercise intensity SmO_2_ at locomotor muscles tends to be lower, reflecting the greater difficulty matching O_2_ delivery to extraction. During incremental cycling exercise, SmO_2_ measured at the vastus lateralis (VL) typically desaturates as a function of increasing exercise intensity, generally following a sigmoidal or segmented linear profile (16–20). The behaviour of SmO_2_ has been studied for both continuous and constant workloads (CW) at various intensities (7–14). With increasing workload, a desaturation is expected for SmO_2_, indicating increased oxygen extraction at the observed tissues (19). SmO_2_ desaturation typically varies monotonically as a function of exercise intensity, generally following a sigmoidal or segmented linear profile (16–20).

During CW cycling exercise, SmO_2_ at the VL will rapidly deoxygenate before reaching a quasi-steady state within 60–120 s, similar to the primary onset phase of systemic V̇O_2_ kinetics (21, 22). At this quasi-steady state, SmO_2_ may gradually rise at lower intensity, and gradually fall at higher intensity (15, 23, 24).

An incremental multi-stage cycling test combines aspects of both incremental ramp and CW exercise in a single protocol. Constant workload stages are performed at progressively increasing workloads up to maximal exercise tolerance. Work steps are ideally 3 min in duration or longer to permit physiological measures to reach a relative steady-state at each workload (25, 26). Between each work step, a 1 min passive recovery interval is added to observe the onset desaturation response of each work step. By employing this protocol, the progressive effects of each workload on all performance and physiological markers can be observed in relative isolation in a time efficient single session (27, 28). Recently, this protocol has been used to compare SmO_2_ with [BLa], using a similar rationale outlined previously (28). Despite the benefits of segmenting each intensity with an integration of passive rest periods for identifying the internal load response of each work step, reliability of SmO_2_ during such a protocol remains unclear.

Multiple studies have validated various stationary NIRS sensors with each of the physiological and performance markers outlined above, during various modes of exercise (12, 17, 22, 29–31). Despite these reports, what portable, wearable NIRS test–retest reliability will be in comparison with each of these markers during a common exercise assessment protocol remains unclear. If wearable NIRS is intended as a commercially available, non-invasive instrument to be used by athletes and coaches to provide useful information about internal load during exercise, addressing this question is of great importance. The purpose of our study was to estimate the reliability of muscle oxygenation, alongside common performance, and physiological markers across exercise intensity, as well as to provide absolute agreement in original units that can be expected between sessions for each marker.

We hypothesized that SmO_2_ measured with the Moxy muscle oxygen monitor at the VL would demonstrate good test–retest reliability across intensity during an incremental multi-stage cycling test incremental protocol (3–5, 16, 28). We also hypothesized that relative reliability scores for SmO_2_ would be lower than for V̇O_2_ and HR, higher than [BLa] and RPE, and have larger thresholds for minimal detectable change compared with V̇O_2_ and HR, but not RPE and [BLa] (32–39).

## 2. Materials and methods

### 2.1. Participants

Twenty-one trained cyclists (10 females and 11 males; 29.1 ± 7.8 years of age, 69.6 ± 11.3 kg, 174 ± 11 cm, 58.6 ± 7.9 ml kg min^−1^ peak oxygen uptake) volunteered and provided written informed consent to participate in this experiment. Descriptive statistics are presented in Table 1. To obtain sufficient power of *β* = 0.8 with *α* = 0.05, an *a priori* sample size calculation was made in G*Power software (version 3.1.9.7, Kiel, Germany) using previously reported data from other groups that compared SmO_2_ values within and between sessions during ramp incremental tests and severe intensity efforts (5, 15, 16, 40, 41). This study was conducted in accordance with the principles established in the declaration of Helsinki and approved by the research ethics committee of The University of British Columbia (H21-00446). The inclusion criteria were healthy athletes with at least 2 years of training experience in cycling, aged 18–48 years, V̇O_2_peak ≥45 ml kg^−1^ min^−1^ for females and ≥50 ml kg^−1^ min^−1^ for males, non-smokers, with no history of cardiovascular disease, and no injuries requiring time away from training within the previous six months (42–44). Sex was classified by self-report and all participants identified as either male or female.

**Table 1:** Baseline characteristics and peak parameters for all participants.

Measurement	Participants (*n* = 21)
Age (years)	29.1 ± 7.8
Weight (kg)	69.6 ± 11.3
Height (cm)	174 ± 11
VL SF (mm)	9.6 ± 4.7
V̇O_2_peak (ml·min^−1^)	4,126 ± 1,062
V̇O_2_peak (ml·kg^−1^·min^−1^)	58.6 ± 7.9
Wpeak (W)	306 ± 79
Wpeak (W·kg^−1^)	4.4 ± 0.6
[BLa]peak (mmol·L^−1^)	13.6 ± 3.5

Data reported as mean ± SD.

### 2.2. Experimental design

Participants visited the laboratory on two occasions one or two weeks apart, at the same time of day (±1 h) to assess test–retest reliability of NIRS parameters during an incremental multi-stage cycling test. Trials were performed outside of the participants' competitive season. Participants were instructed to avoid strenuous exercise for 24 h (hrs), avoid alcohol for 12 h, avoid caffeine for 4 h, maintain the same diet for 12 h, and get at least 8 h of sleep prior to both trials.

At the start of the first trial, participants' height and mass were recorded, and skinfold measurements (Harpenden Skinfold Caliper, Baty International, West Sussex, England) were taken at the right vastus lateralis at the placement of the NIRS sensor (detailed below).

Each trial consisted of an incremental multi-stage cycling test, with 5 min CW stages, each followed by a 1-min passive rest interval. The protocol began at 1.0 W kg^−1^ and progressed by 0.5 W kg^−1^ per stage. Participants performed the first trial at a self-selected cadence and maintained ±5 cadence range throughout the second trial. Participants performed the first trial to maximal exercise tolerance or until cadence fell by more than 10 rpm for 10 s, despite strong verbal encouragement. This cutoff was selected to minimize the effect of changing cadence on SmO_2_ (45). In the second trial, the protocol was repeated to the same duration as the first trial (*n* = 18) or to the limits of tolerance if they were unable to reach the same end point (*n* = 3). Participants maintained a seated position with both hands on the handlebars in their preferred riding position for the entire exercise protocol.

### 2.3. Data collection

The cycling protocol was performed on the participant's own bicycle mounted to an electronically controlled stationary trainer (Tacx NEO 2T, Garmin International Inc., Olathe, KS, USA). Resistance was controlled and cycling power output (watts) and cadence [revolutions per minute (rpm)] were recorded at 1 Hz using PerfPRO Studio Software (Hartware Technologies, Rockford, MI, USA) installed on a laptop computer.

Heart rate [beats per minute (bpm)] was recorded from a chest strap monitor (Garmin International Inc., Olathe, KS, USA) at 1 Hz. Capillary [BLa] (mmol·L^−1^) was sampled from the fingertip during the last 30 s of each work stage and measured with an electrochemical biosensor analyzer (Edge USA, USA). RPE was reported at the same time using the Borg category-ratio 0–10 scale (46). Expired gases were collected and sampled by an open circuit metabolic analyzer (TrueOne 2400, ParvoMedics Inc., Sandy, UT, USA) from a 4 L mixing chamber and V̇O_2_ was recorded as 15 s average values.

Muscle oxygen saturation was measured at the right vastus lateralis (VL) using a wearable NIRS sensor (Moxy Monitor, Fortiori Design LLC., Hutchinson, MN, USA). The Moxy monitor is a self-contained, relatively low cost, wearable continuous wave NIRS sensor that can resolve an arbitrarily scaled heme volume that represents both hemoglobin and myoglobin concentration in the tissue under illumination, as well as SmO_2_ on a 0%–100% scale (4). The sensor was positioned on the right VL muscle belly at ⅓ the distance from the patella to greater trochanter with the participant seated with knee bent to 90°. The sensor was secured with adhesive tape along with the manufacturer-supplied light shield to minimize signal interference from ambient light and movement.

Moxy employs four wavelengths of near-infrared light (680, 720, 760, and 800 nm), with a single LED source and two detectors at 12.5 and 25 mm separation, giving a maximum penetration depth of approximately 12.5 mm (3). It is recommended that participant skinfold thickness (SF) be less than the maximum penetration depth (SF < ½ the maximum inter-optode distance); however, to better represent real-world use of NIRS with competitive athletes, no exclusion was made on the basis of skinfold measurements, nor was a correction performed for SF (47). The SmO_2_ signal was used as the primary muscle oxygenation variable in this study (3–5). SmO_2_ was recorded every 2 s (0.5 Hz) and smoothed with a 5 s moving average as per manufacturer default settings.

### 2.4. Data analysis

V̇O_2_peak was determined as the highest two consecutive 15 s measurements within each trial. Peak workload (Wpeak) was determined for each trial using the following formula:

$$\mathrm{Wpeak}=WC\frac{WC+\;\Delta W\cdot t}{300}$$

Where *WC* is the workload of the prior completed stage, Δ*W* is the final incremental workload (0.5 *W* kg^−1^), and *t* is the time completed at the final work stage. HRpeak was determined as the highest 1 s value recorded during each trial.

To evaluate test–retest reliability of each parameter across exercise intensity between the two trials, we analyzed the first, median, and last work stages performed by each participant. These will be referred to as lowest, median, and highest workloads. Continuous variables (SmO_2_, V̇O_2_, and HR) were averaged from the last 60 s within each work stage, omitting the final 10 s to exclude influence from the end of work stage transition to rest.

During analysis, three sets of measurements were excluded due to measurement error or methodological inconsistencies. First, gas exchange measurements for two participants were excluded. All V̇O_2_ data from one participant was excluded while the data from the final stage of the other was excluded. Second, technical issues with the ergometer resistance occurred for two participants, which affected the lowest and median work stages, respectively. These issues led to the [BLa], V̇O_2_, and SmO_2_ data having to be discarded. Lastly, the HR signal during the first stage in one participant was not acquired.

### 2.5. Statistical analysis

Data analysis and statistical analysis were carried out in R (v4.1.2, R Foundation for Statistical Computing, Vienna, Austria). Descriptive results are presented as mean ± standard deviation (SD) or 95% confidence interval (CI95%), as indicated. A two-way repeated measures ANOVA was performed to evaluate the effects of workload (lowest, median, highest) and trial (1, 2) on each variable. Pairwise *post hoc* comparisons were made to evaluate for a main effect of trial and adjusted for multiple comparisons with the Bonferroni method. Significance was set at *p* < 0.05. Normality was assessed by Shapiro–Wilk tests.

Relative reliability was quantified as intraclass correlation coefficient (ICC_2,1_) (2, 6). Interpretations of poor (<0.5), moderate (0.5–75), good (0.75–0.9), and excellent (>0.9) were used (48).

Absolute reliability and within-subject agreement were quantified as the SEM in original units, which is appropriate for homoscedastic data (2, 49, 50). All outcome variables evaluated in the present study were found to be homoscedastic from visual analysis and Levene's test, except for [BLa]. Absolute reliability was also quantified as a coefficient of variation (CV) from within-subject repeated measurements (Atkinson & Nevill, 1998). The minimal detectable change (MDC) was calculated from SEM at a CI95% (Hopkins 2000; Weir 2005), as:

MDC=SEM×1.96×√2.

## 3. Results

### 3.1. Test–retest comparisons

**Figure 1:**
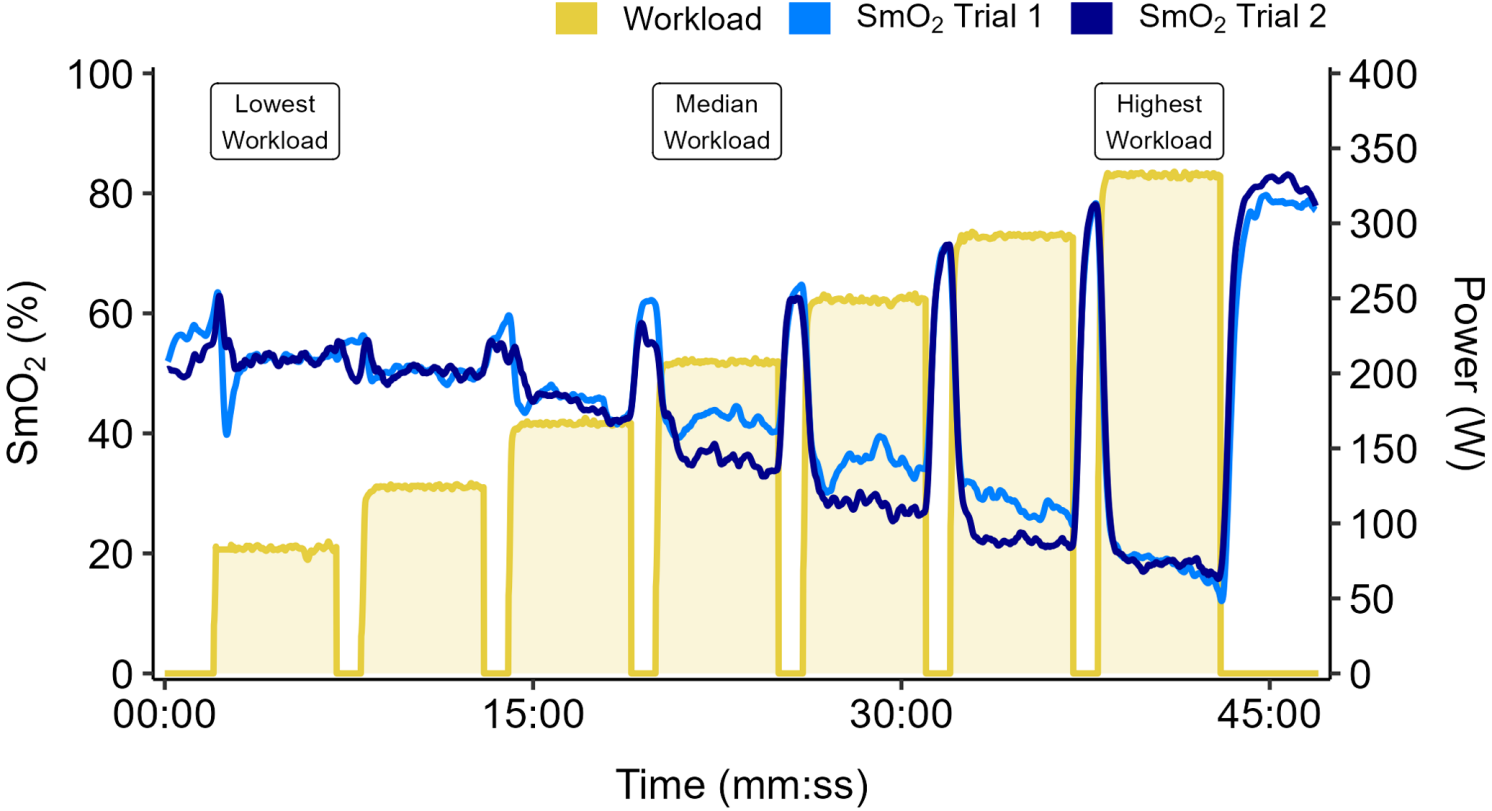
Data shown for a representative participant performing two trials of an incremental multi-stage cycling test. Work stages are 5 minutes with 1-min passive rest intervals. Workload starts at 1.0 W·kg^−1^ and increases by 0.5 W·kg^−1^ each stage. Muscle oxygen saturation (SmO_2_) overlaid from Trial 1 (light blue) and Trial 2 (dark blue). Participants performed the first trial to the maximal limits of tolerance. The second trial was repeated to the same duration, or to the limits of tolerance if they were unable to reach the same duration as the first trial.

**Figure 2:**
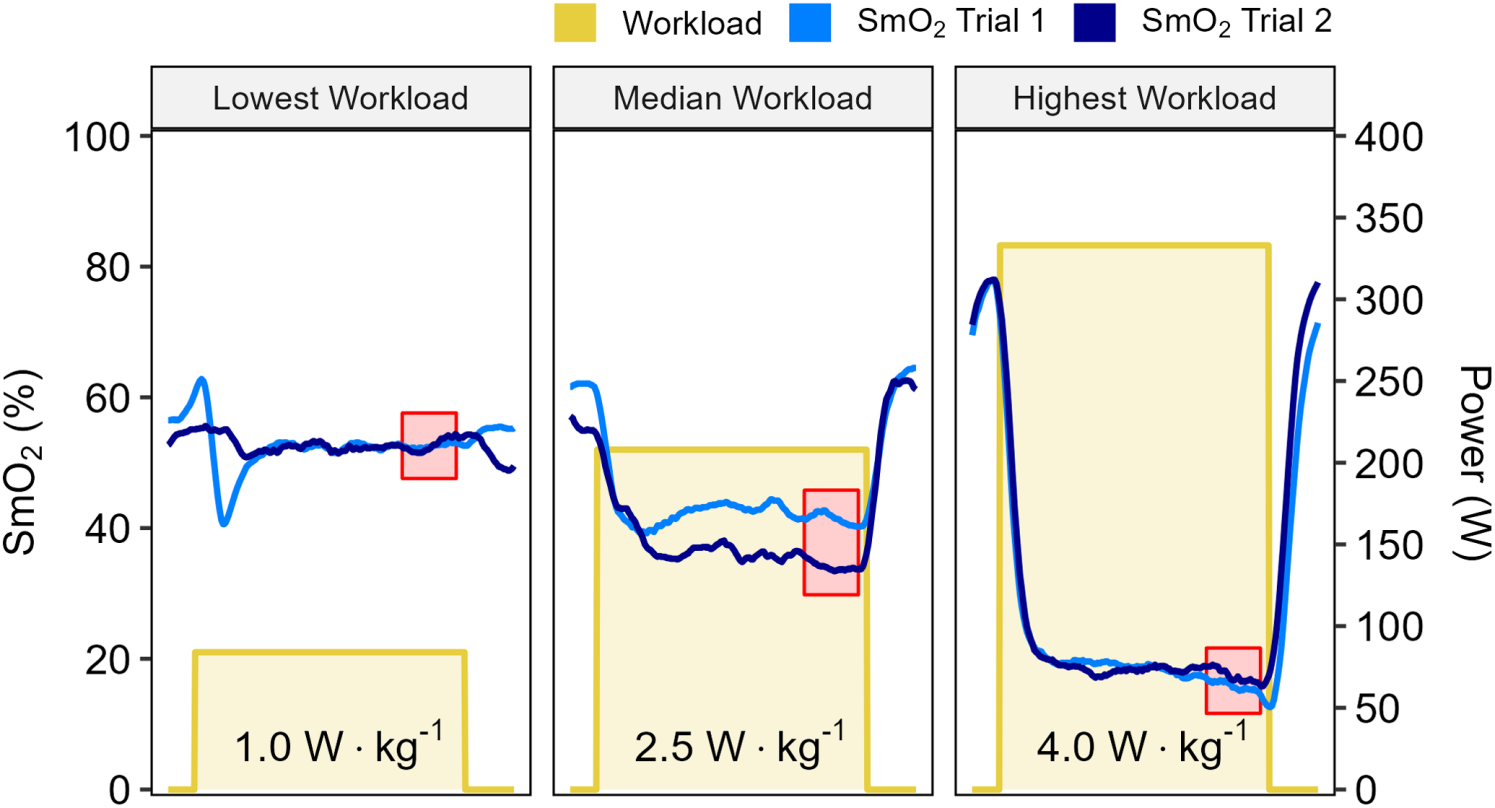
Data shown for a representative participant at the lowest, median, and highest work stages from two trials, with workloads displayed in W·kg^−1^. Muscle oxygen saturation (SmO_2_) recorded from Trial 1 (light blue) and Trial 2 (dark blue). Mean SmO_2_ data from the last 60 seconds of each work stage was used for test-retest comparisons (red shaded areas).

**Figure 3:**
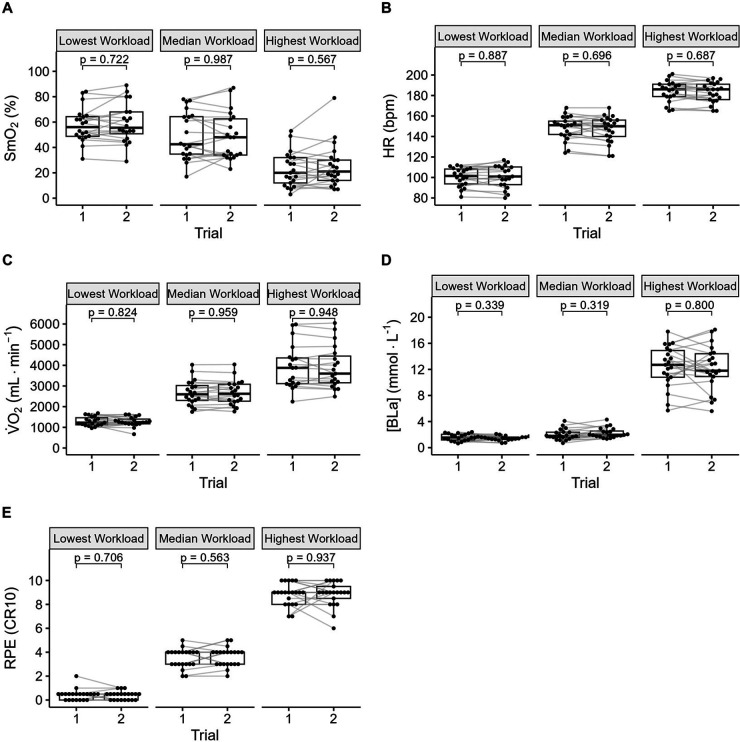
Between trial comparisons of (**A**) muscle oxygen saturation (SmO_2_), (**B**) heart rate (HR), (**C**) systemic oxygen uptake (V̇O_2_), (**D**) concentration of blood lactate ([Bla]), and (**E**) rating of perceived exertion (RPE).

Data from a representative participant performing the full incremental multi-stage cycling protocol, with muscle oxygen saturation (SmO_2_) response from two trials are displayed in Figure 1. Data from the same participant at the lowest, median, and highest workloads are displayed in Figure 2. Descriptive group results for trials 1 and 2 can be found in Table 2. Test-retest comparisons between trials at the lowest, median, and highest workloads are displayed in Figure 3. Between-trials comparisons showed no significant differences within each workload for all outcome variables. Workload at the lowest, median, and highest stages were 71 ± 11, 199 ± 47, and 303 ± 80 W, respectively. Cadence showed an effect of workload between lowest and median (*p* < 0.01), and lowest and highest workloads (*p* < 0.001), with no difference between median and highest (94 ± 8) workloads (*p* = 0.062) (Table 2).

**Table 2:** Performance and physiological parameters for the lowest, median, and highest workloads (mean ± SD).

Parameter	Measurement	Lowest	Median	Highest
SmO_2_ (%)	*n*	20	20	21
	Grand mean	58 ± 14^a^^,^^b^	49 ± 18^c^	24 ± 15
	Trial 1	58 ± 14	49 ± 19	23 ± 14
	Trial 2	59 ± 15	49 ± 19	25 ± 17
	Mean diff	2 ± 6	0 ± 9	3 ± 9
HR (bpm)	*n*	20	21	21
	Grand mean	101 ± 10^a^^,^^b^	148 ± 12^c^	184 ± 10
	Trial 1	101 ± 9	148 ± 12	184 ± 10
	Trial 2	101 ± 11	147 ± 13	183 ± 10
	Mean diff	0 ± 4	1 ± 6	1 ± 4
V̇O_2_ (ml·min^−1^)	n	19	20	19
	Grand mean	1,285 ± 225^a^^,^^b^	2,677 ± 585^c^	3,904 ± 1,033
	Trial 1	1,294 ± 224	2,672 ± 599	3,915 ± 1,067
	Trial 2	1,277 ± 232	2,682 ± 585	3,893 ± 1,027
	Mean diff	17 ± 170	10 ± 148	22 ± 255
[BLa] (mmol·L^−1^)	*n*	20	20	21
	Grand mean	1.5 ± 0.5^b^	2.1 ± 0.8^c^	12.3 ± 3.3
	Trial 1	1.6 ± 0.5	2.0 ± 0.8	12.4 ± 3.2
	Trial 2	1.4 ± 0.4	2.2 ± 0.8	12.2 ± 3.4
	Mean diff	0.1 ± 0.5	0.3 ± 0.6	0.3 ± 2.4
RPE (CR10)	*n*	21	21	21
	Grand mean	0.4 ± 0.4^a^^,^^b^	3.5 ± 0.8^c^	8.8 ± 1.0
	Trial 1	0.4 ± 0.5	3.5 ± 0.8	8.8 ± 0.9
	Trial 2	0.4 ± 0.4	3.6 ± 0.8	8.9 ± 1.0
	Mean diff	0.0 ± 0.4	0.1 ± 0.6	0.0 ± 1.2

The number of participants (n) with data from two trials used for test-retest comparisons at lowest, median, and highest workloads with mean Trial 1 and 2, mean of both trials together (Grand mean) and difference between trials (Mean diff). Muscle oxygen saturation (SmO_2_), heart rate (HR), systemic oxygen uptake (V̇O_2_), concentration of blood lactate ([BLa]), and rate of perceived exertion (RPE).

^a^ Indicates significant differences between lowest and median work stages.

^b^ Indicates significant differences between lowest and highest work stages.

^c^ Indicates significant differences between median and highest workloads. There were no significant differences between trials.

### 3.2. Relative reliability

ICC for SmO_2_ were good to excellent. ICC for HR and V̇O_2_ were good to excellent, apart from V̇O_2_ at the lowest workload, which was moderate. ICC for [BLa] and RPE were poor to moderate (Table 3).

**Table 3:** Relative reliability is reported using ICC scores, CV, SEM, and MDC.

Parameter	Measurement	Lowest	Median	Highest
SmO_2_ (%)	ICC	0.90 (0.77–0.96)	0.90 (0.76–0.96)	0.81 (0.59–0.92)
	CV	11.0% (8.4–16.1%)	22.9% (17.4–33.5%)	43.8% (33.5–63.3%)
	SEM	5 (3–7)	6 (4–9)	7 (5–10)
	MDC	12	16	18
HR (bpm)	ICC	0.91 (0.79–0.96)	0.87 (0.71–0.94)	0.92 (0.81–0.97)
	CV	4.2% (3.2%–6.2%)	4.7% (3.6%–6.7%)	2.6% (2.0%–3.7%)
	SEM	3 (2–4)	4 (3–6)	3 (2–4)
	MDC	8	12	8
V̇O_2_ (ml·min^−1^)	ICC	0.73 (0.42–0.89)	0.97 (0.93–0.99)	0.97 (0.93–0.99)
	CV	13.1% (9.9–19.4%)	6.4% (4.9%–9.4%)	6.5% (4.9%–9.6%)
	SEM	116 (88–172)	101 (77–148)	174 (131–257)
	MDC	323	280	482
[BLa] (mmol·L^−1^)	ICC	0.44 (0.03–0.73)	0.71 (0.40–0.88)	0.74 (0.47–0.89)
	CV	39.3% (29.9–57.4%)	35.7% (27.1–52.1%)	22.8% (17.4–32.9%)
	SEM	0.3 (0.3–0.5)	0.4 (0.3–0.6)	1.7 (1.3–2.4)
	MDC	0.9	1.2	4.6
RPE (CR10)	ICC	0.64 (0.29–0.83)	0.70 (0.40–0.86)	0.29 (−0.17–0.64)
	CV	16.8% (11.7–29.5%)	16.5% (12.6–23.8%)	12.2% (9.3–17.6%)
	SEM	0.2 (0.2–0.4)	0.4 (0.3–0.6)	0.8 (0.6–1.2)
	MDC	0.7	1.2	2.2

95% Confidence intervals in brackets. Coefficient of variation (CV) units are in percent. Absolute reliability is reported as standard error of the measurement (SEM) and minimal detectable change (MDC) use the original units of each parameter. Muscle oxygen saturation (SmO_2_), heart rate (HR), systemic oxygen uptake (V̇O_2_), blood lactate concentration ([BLa]), and rate of perceived exertion (RPE).

### 3.3. Absolute reliability

SEM (95% CI) for SmO_2_ was 5 (3–7), 6 (4–9), and 7 (5–10)%, and MDC was 12%, 16%, and 18% at the lowest, median, and highest workloads, respectively (Table 3).

## 4. Discussion

The aim of this study was to estimate and compare the reliability of wearable NIRS with common performance and physiological measures across an incremental multi-stage cycling test on a stationary cycling ergometer. Our results for test–retest analysis supported our hypothesis that SmO_2_ would demonstrate good reliability across intensity, with excellent ICC scores at the lowest and median workloads, and a good ICC at the highest workload. With regards to relative reliability compared with the remaining variables, SmO_2_ provided similar ICC values to both HR and V̇O_2_, scoring higher than V̇O_2_ at the lowest workload, but not as high as both of these variables at the highest workload. Compared with [BLa] and RPE, SmO_2_ had better ICC scores across intensity (Table 3). This suggests that for use as an everyday tool for monitoring training load, SmO_2_ is similarly sensitive to physiological variability as these other measures.

Compared to previous studies of reliability of wearable NIRS (3–5), our results demonstrated higher absolute agreement compared to the studies that examined the reliability of wearable NIRS at rest. These studies reported absolute agreement as a CV of approximately 5% SmO_2_ (2.5%–9.5%) (3), or using Bland-Altman limits of agreement of approximately 10% SmO_2_ between trials (4), versus our CV of 11%–44% SmO_2_ from lowest to highest intensity respectively. However, we observed that SmO_2_ was homoscedastic, with a similar variability at high and low values (at low and high intensity, respectively). CV is less appropriate for homoscedastic data where variance is not proportional to parameter values, such as for SmO_2_. However, its common use in sport science reliability studies justified its inclusion (2, 49, 50). Caution is advised with the interpretation of CV for SmO_2_ in this context. Our ICC scores across workloads were comparable to those by Crum et al., who also looked at the reliability of wearable NIRS during an incremental exercise test (IET) (5). Like our CV results, they found higher CV values (lower absolute reliability) as workload increased. It is worth considering that the direction of change for SmO_­2_ alone is inversely related to intensity, while all other parameters increase along with intensity. Our advice for sport practitioners is to consider the absolute limits of agreement when interpreting test–retest values. This can be quantified by the SEM when considering daily variation, and the MDC for interpreting the results of a longitudinal training intervention.

A study by van Hooff et al., investigated the reliability of PortaMon, wearable NIRS sensor during a continuous IET (51). They reported less variability with CV scores of 6, 8, and 10% at baseline, post exercise recovery, and maximal task tolerance, respectively. Despite the lower variability, their relative reliability scores were lower than the ones detected in our results. Unlike the Moxy sensor used in our study, the PortaMon sensor is more expensive, offering greater illumination depth and sampling rate. A study by McManus et al., compared between the PortaMon and Moxy during rest, exercise, and arterial occlusion (3). They found that Moxy displayed a greater dynamic range during exercise and arterial occlusion. Therefore, it is possible that the differences in both absolute and relative reliability between van Hooff's group and our results is explained by sensor differences.

Our results in Table 3 provide useful information for athletes, practitioners, and sport scientists who wish to monitor changes in VL SmO_2_ over time using a Moxy wearable sensor. Having SEM and MDC quantified across intensity can aid in understanding the expected day to day variance for SmO_2_ during regular training and testing. Changes within a range of ±SEM may be meaningful for an individual athlete but should not be considered to represent a significant difference. A longitudinal intervention such as a structured training program can be considered to have a significant effect on SmO_2_ if the values measured at a given absolute workload change by more than the MDC. Additionally, having all other common measures presented in conjunction with SmO_2_ improves our understanding of what each expected change should be for specific exercise workloads over time.

### 4.1. SmO_2_ compared to V̇O_2_ and HR

As expected, V̇O_2_ presented excellent reliability at all but the lowest workload, potentially due to a signal noise ratio issue. Since the early 1980’s, V̇O_2_ has been shown to demonstrate good-to-excellent test–retest reliability scores with a CV of approximately 10% across intensity during incremental exercise tests under controlled conditions (35, 37). In a recent investigation on the reliability of V̇O_2_ during an IET, Pallarés et al. (2016), observed the reliability of V̇O_2_ associated with the gas exchange threshold and the respiratory compensation point (37). These are common thresholds used to demarcate the transitions from moderate to heavy and heavy to severe intensity domains, respectively. They reported excellent test–retest ICC scores for both gas exchange threshold and the respiratory compensation point. In their report, within-subject reliability for both thresholds was found to have slightly lower CV (3.6% and 2.1%, respectively) than those found in our study at the median and highest workload (6.4% and 6.5%) (37).

In another study, reliability of V̇O_2_ and ventilatory measurements were estimated for the ParvoMedics TrueOne 2400 metabolic cart during a cycling ergometer IET (52). The protocol included 10–12 min work steps with 50 W increments, starting at a resistance of 50 W and progressing to 250 W. For V̇O_2_, their results showed a CV of approximately 5%, providing further strength to our findings, especially during the highest workload.

As for HR, Montoye et al. observed the reliability of HR using a chest strap during an IET. The protocol included 3 min work steps with 50 W increments, starting at a resistance of 50 W to maximal task tolerance. Their results showed excellent test–retest reliability scores, with CV less than 5% as seen in our results (36). In our results, HR showed CV ranging from 2.6% to 4.7% across intensity, and good to excellent ICC scores, which was higher than SmO_2_ only at the highest workload.

As expected, both V̇O_2_ and HR did show better reliability compared with SmO_2_, but only during the highest intensity. Despite difference in the mechanisms related to each measurement, comparing them side-by-side during an incremental exercise test provides an interesting contrast to better understand how reliable SmO_2_ is between sessions, in reference to these two common measures.

### 4.2. SmO_2_ compared to RPE and [BLa]

Our results showed higher reliability for SmO_2_ compared to both RPE and [BLa] across intensity. Both RPE and [BLa] ICC scores were either poor or moderate, compared with the good to excellent ICC scores for SmO_2_. Both of these measures have shown a wide range of reliability scores during incremental exercise tests (32–34, 36, 37). In our study, RPE ICC scores were poor-to-moderate, with ICC at the highest workload demonstrating the lowest ICC score across all outcome variables. As for [BLa], ICC scores were poor at the lowest workload, and moderate at the median and highest workloads. Our findings were in agreement with previous reports by Ettema et al., that showed variable test–retest scores for [BLa], across similar intensities (ICC = 0.44–0.92, MDC = 26%–69%) (38). It is worth highlighting that both [BLa] and RPE were taken using single measurements at the end of each stage, unlike the other continuous measures. They are also more prone to subjective interpretation of RPE scores, and investigator error in accurately acquiring capillary blood samples during exercise (34, 53). Despite [BLa] and RPE measuring different mechanisms, they are commonly used in exercise testing. Thus, contrasting them with SmO_2_ aids in highlighting how reliable each of these measures are in this context.

## 5. Study limitations

First, our experimental design did not include a familiarization trial to limit participant burden, which may have negatively affected the test–retest reliability outcomes. As previously mentioned, RPE scales are known to require participant familiarization periods, to ensure accurate self-reporting of exercise exertion (53). They are also dependent on other external factors such as psychological stress, emotional state, and readiness (53). As such, it is possible that the lack of familiarization negatively impacted the RPE reliability outcomes compared with the other measures used in our study. Second, the SmO_2_ signal of the wearable, self-contained Moxy sensor may be more sensitive to changes in blood flow, and changes in tissue properties with small positioning inconsistencies compared to NIRS signals measured with more advanced stationary technologies (21, 47, 54, 55). These sensor limitations may have affected the quality of the signal, and as a result, the test–retest results presented in our study. In order to include a more representative sample size of healthy, trained male and female cyclists, we did not exclude any participants based on skinfold thickness, even though previous reports suggest signal quality may be affected by skinfold thickness greater than 12.5 mm (3, 4, 56). Our rationale for providing a more generalizable subject group was a compromise that possibly affected reliability outcomes. And third, as muscle recruitment is specific to exercise modalities, NIRS responses will be specific to the location and modality (57, 58). Other locations may demonstrate higher physiological variation, although the measurement uncertainty of the Moxy device itself may be consistent across modalities (59).

## 6. Conclusions

Our main objectives were estimating the reliability of muscle oxygen saturation, alongside common performance and physiological markers across exercise intensity, as well as to provide absolute agreement in original units that can be expected between sessions for each marker. Our findings show that the commercially available Moxy wearable NIRS sensor provides good-to-excellent test–retest reliability during an incremental multi-stage cycling protocol. Compared to other common physiological metrics, reliability was highest for V̇O_2_ and HR, followed by SmO_2_, with [BLa] and RPE having lower reliability. Additionally, estimated values of absolute agreement reported in our results provide practitioners with the ability to differentiate measurement error from “true” physiological change. Athletes and coaches should expect a certain range of day to day variability when assessing and monitoring performance. This knowledge can be applied by practitioners who use this non-invasive, affordable technology for training prescription or monitoring internal load alongside other common measures. Wearable NIRS is an important instrument that if used appropriately, can add valuable insight into understanding muscle metabolic responses to exercise.

## Data Availability

The raw data supporting the conclusions of this article will be made available by the authors, without undue reservation.
